# Catastrophic Wear of an Acetabular Component Misdiagnosed as Total Hip Arthroplasty Dislocation

**DOI:** 10.7759/cureus.11672

**Published:** 2020-11-24

**Authors:** Ioannis Papaioannou, Georgia Pantazidou, Stamatia Chatziperi, Andreas Baikousis, Panagiotis Korovessis

**Affiliations:** 1 Orthopedics and Traumatology, General Hospital of Patras, Patras, GRC; 2 Otolaryngology - Head and Neck Surgery, General Hospital of Patras, Patras, GRC

**Keywords:** acetabular shell, wear, dislocation, metallosis, failure, polyethylene

## Abstract

Total hip arthroplasty constitutes the operation of the century, although not without complications, which require revision surgery due to loosening, infection, dislocation, and wear. Hereby, we report a rare case of acetabular shell wear misdiagnosed as a dislocation. Patients who underwent total hip arthroplasty with ultra-high molecular weight polyethylene are more vulnerable to excessive wear, and close monitoring can prevent this catastrophic sequence. Timely and accurate diagnosis is mandatory to avoid any unnecessary interventions, such as useless reduction attempts. An anteroposterior radiograph is valuable, although computed tomography can settle the diagnosis with accuracy. Evaluation of any previous radiographic examination is very helpful to highlight any differences. Metal debris shown in the joint space, the bubble sign, and also the eccentric location of the prosthetic head are very helpful signs of the catastrophic wear presented to the X-rays. Since late onset dislocations are rare, orthopedic surgeons should be aware that catastrophic wear of the polyethylene and subsequently the acetabular shell can be presented as a late onset dislocation or protrusion. Furthermore, arthroplasty surgeons should adequately monitor patients who underwent hip arthroplasty with this particular polyethylene type.

## Introduction

There is substantial evidence to indicate that total hip replacement (THR) effectively reduces pain and improves patients' function with primary or secondary hip osteoarthritis. This is the reason why this surgical intervention has been characterized as the operation of the century [1]. For primary THR, an increase of 174% is estimated in the United States by 2030 [2]. This huge increase also has a crucial impact on cases of THRs, which require revision surgery. Total hip revisions are projected to grow by 137% until 2030 [2]. The cases of THR, which commonly require revision surgery, are dislocation, component wear, infection, and prosthesis loosening [3]. Hereby, we report a rare case of acetabular wear misdiagnosed as a dislocation. Catastrophic wear of total hip arthroplasty's components can be easily misdiagnosed as dislocation, and orthopedic surgeons should be aware. Furthermore, we appose diagnosis criteria and propose methods to avoid this devastating complication.

## Case presentation

**Figure 1:**
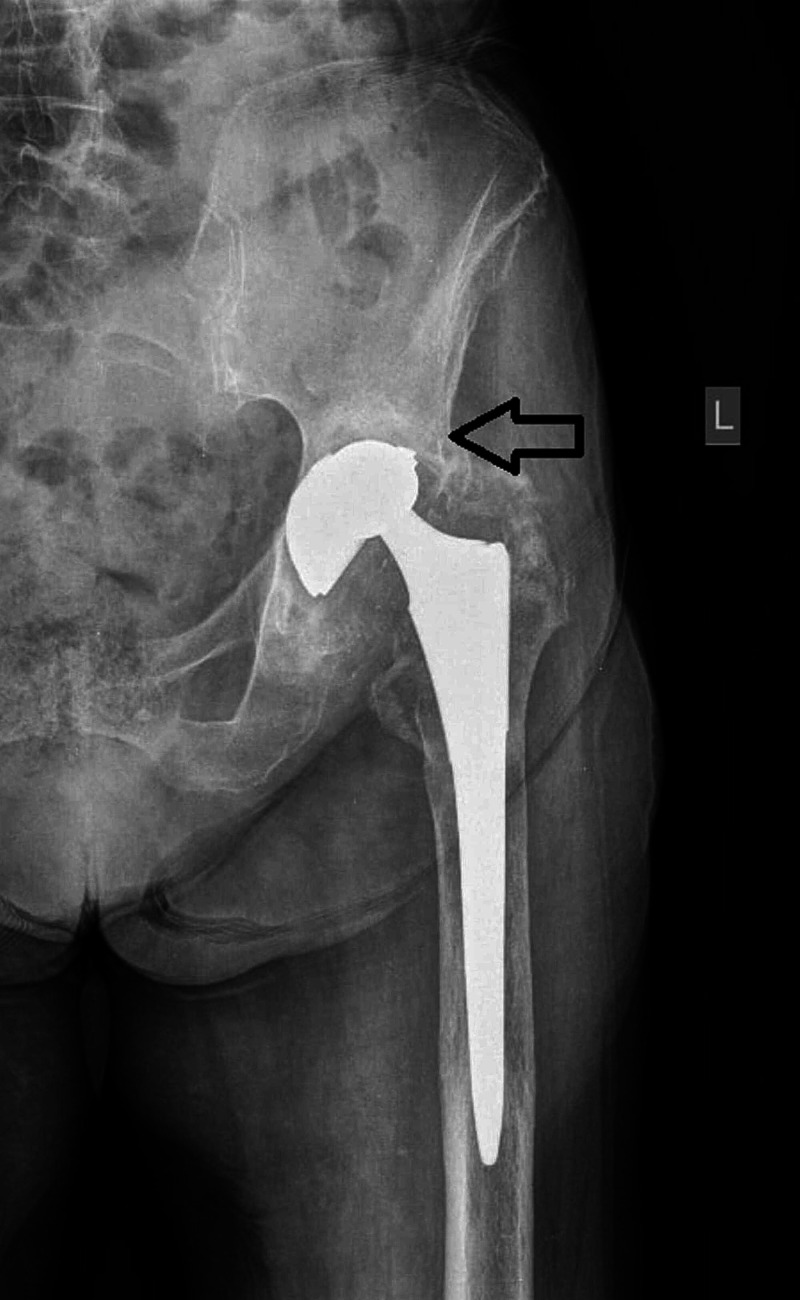
Anteroposterior X-ray of left hip arthroplasty on admission The black arrow shows the “unusual” dislocation of the joint.

An 81-year-old woman was admitted to the authors' emergency department complaining of left hip pain and weight-bearing inability. Mild pain was present for at least six months ago; however, there was a significant deterioration in the last week. Before this time frame, the patient was actually asymptomatic, without any history of trauma or fall in this period. A cementless total hip arthroplasty (Interfit cup and Synergy stem, Smith & Nephew, Memphis, USA) was implanted 17 years ago via an anterolateral approach, while the bearing surfaces were metal on polyethylene (ultra-high-molecular-weight polyethylene, not cross-linked). Clinical evaluation revealed no neurovascular deficit, although leg length discrepancy was identified. Excessive pain was also present during passive hip movements and the rolling test. Plain radiographs demonstrated findings that were interpreted as a dislocation (Figure 1).

**Figure 2:**
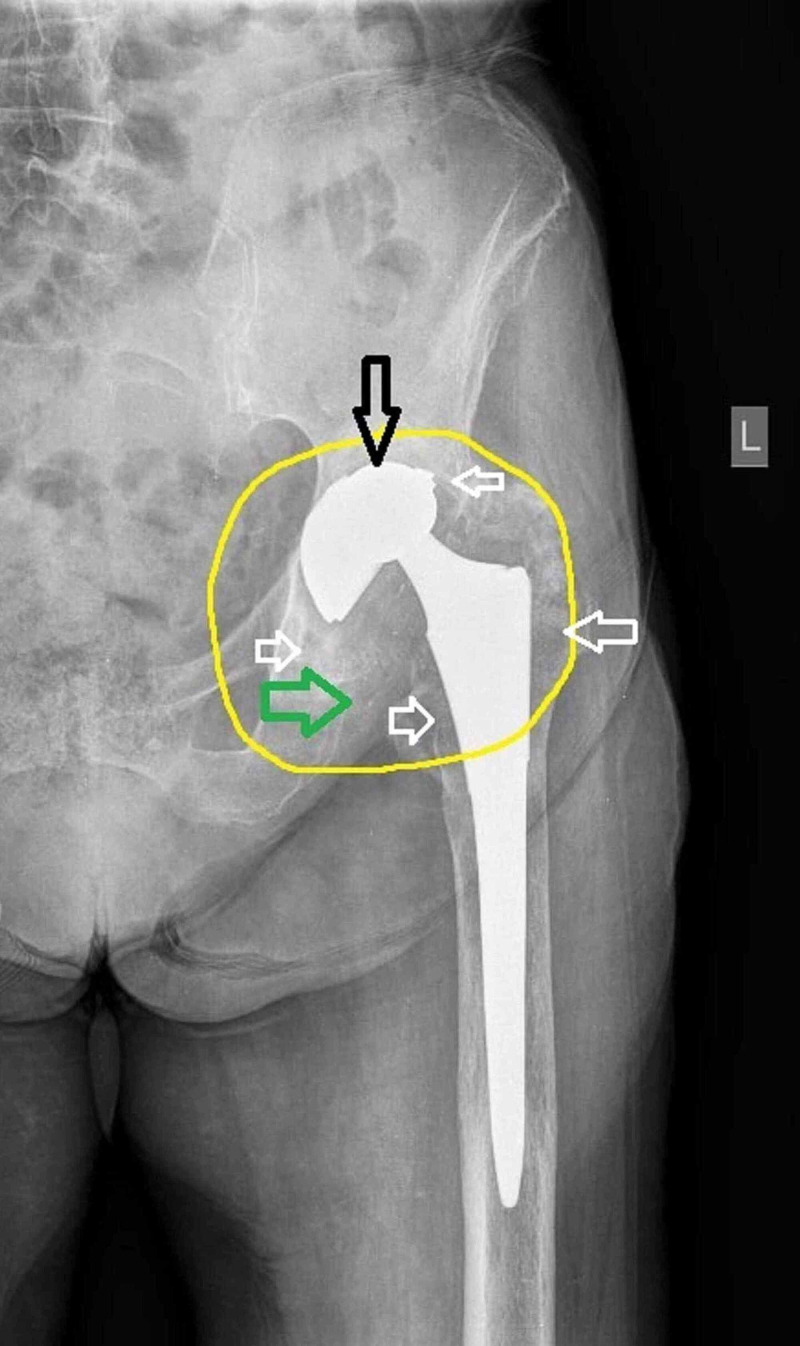
Anteroposterior X-ray of left hip arthroplasty The green arrow shows the metal debris; the yellow circle shows the “bubble sign”; the black arrow shows the erosion of the acetabular component and also the eccentric location of the femoral head, while the small white arrows indicate the areas of bone absorption.

Based on the radiographic evaluation, the patient underwent two attempts for a closed reduction under sedation, without success. A more detailed examination of the anteroposterior view raised the suspicion of a catastrophic failure of the acetabular shell. These findings were the metal debris shown in the joint space, the bubble sign (indicates the enlargement of the joint cavity), the eccentric location of the prosthetic head, the identification of the metal femoral head through the acetabular component, and the bone destruction due to the debris (Figure 2).

**Figure 3:**
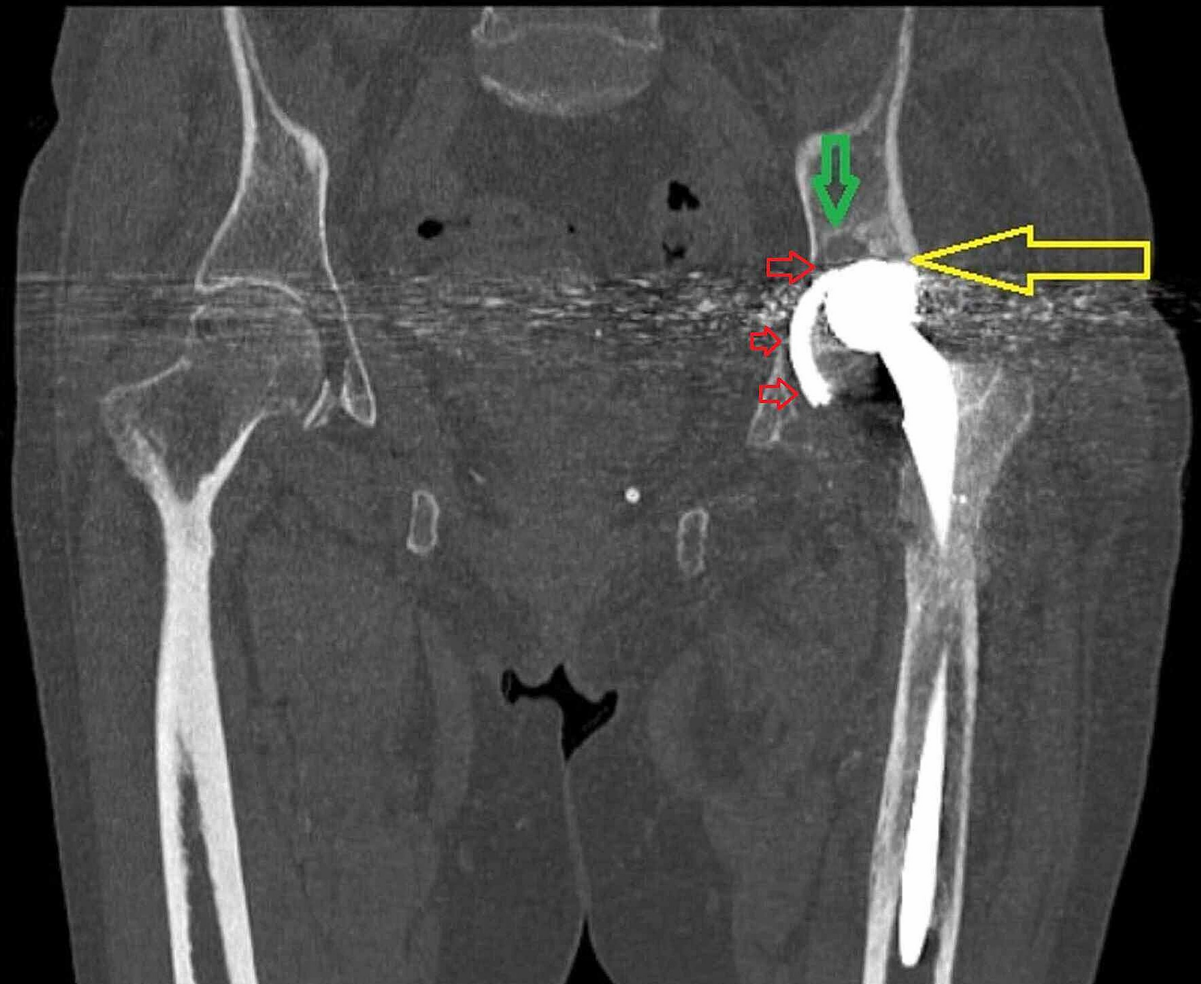
Coronal computed tomography image of the affected hip joint The yellow arrow indicates the protrusion of the femoral head through the acetabular shell; the green arrow demonstrates the focal osteolysis in Delee and Charnley zone 1, and the small red arrows indicate the radiolucent line around the acetabular component.

**Figure 4:**
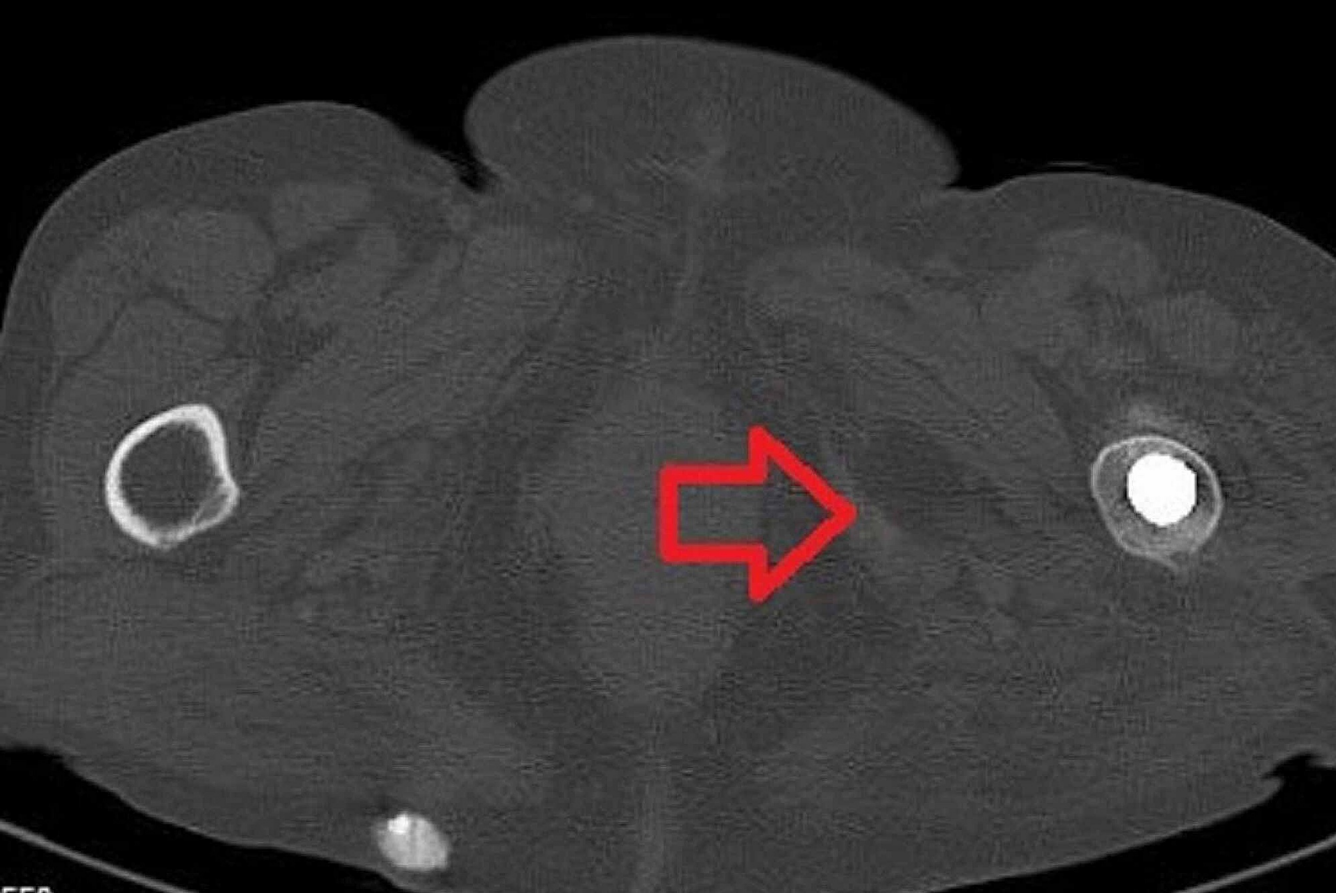
Axial computed tomography image of the affected hip joint The red arrow indicates the cyst formation beneath the adductors muscles.

The patient's erythrocyte sedimentation rate (ESR) and C-reactive protein (CRP) were within normal limits, and the cultures of the synovial fluid aspiration were also negative. According to the radiographic and computed tomography evaluation, a radiolucent line was found around the acetabular shell, while focal osteolysis was confirmed in Delee and Charnley zone 1. Furthermore, the computed tomography confirmed the acetabular component wear and revealed a severe bone loss to the acetabulum (Figure 3) and a cyst formation beneath the adductors muscles (Figure 4).

**Figure 5:**
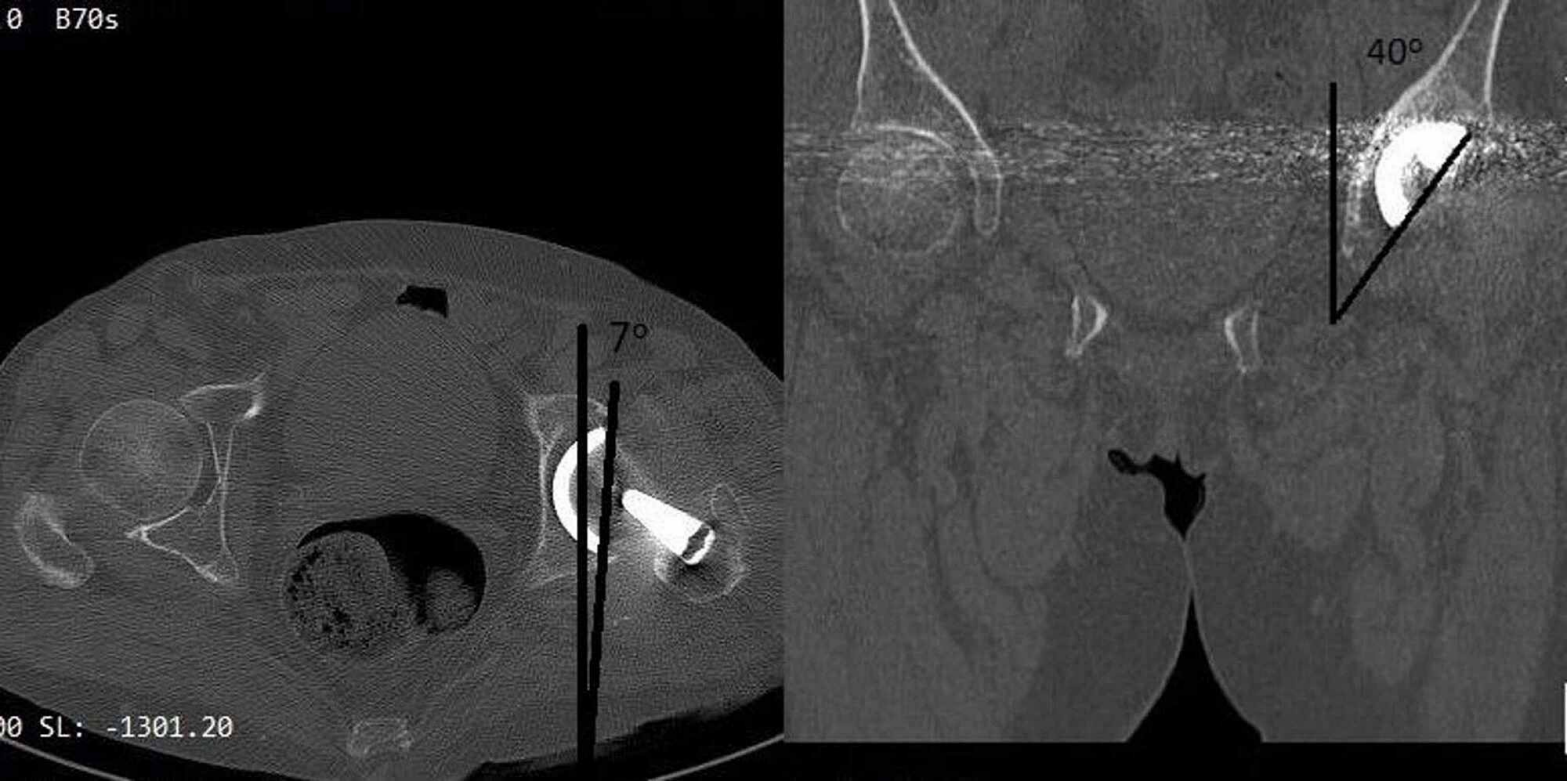
The left axial computed tomography image demonstrates the seven degrees of acetabular shell anteversion, while the right coronal computed tomography image indicates the 40 degrees of abduction

The acetabular shell orientation was recorded (seven degrees of anteversion and 40 degrees of inclination) based on the images of the computed tomography (Figure 5).

The patient was counseled for revision surgery, but her general medical status (recent myocardial infarction) and her chronic diseases (diabetes mellitus, hypertension, and atrial fibrillation) led her to the decision to postpone this major surgery. Until now (six months after the admission to our department), she ambulates with two crutches, and she complains for periods with severe pain, which requires strong painkillers and limitation of mobilization.

## Discussion

Total hip arthroplasty dislocation is a common complication, especially in the early postoperative period, with a reported incidence of 1.1%-4.76% [4]. On the contrary, the incidence of a chronically dislocated prosthesis is rare and increased suspicion is needed in such cases. It is worth noting that the incidence of total hip replacement dislocation is only 0.92% after the second year of the surgical intervention [5]. The published cases in the current literature with a chronic total hip replacement dislocation are less than ten, so increased awareness is needed before settling such a diagnosis [4]. The evidence-based risk factors for total hip arthroplasty dislocation are the increased age, rheumatoid arthritis, femoral head less than 28 mm, and posterior surgical approach [6]. The main causes for late onset hip arthroplasty instability concern polyethylene wear, malposition of the arthroplasty components, neurological deterioration, and dysfunction of the hip abductors [7]. In our case, there was no neurologic deficit in our patient, so the late onset instability was probably due to the conventional polyethylene wear. Polyethylene can cause instability either due to locking mechanism failure or due to wear. Failure of the locking mechanism can result in dislocation of the arthroplasty, while the contact of the femoral head to the acetabular implant can also result in catastrophic wear of the arthroplasty components. On the other hand, the wear of the conventional, not highly cross-linked polyethylene (HXLPE) is the commonest cause of polyethylene failure, which substantially leads to acetabular component wear and catastrophic failure of the hip arthroplasty [8]. In our case, the patient denied further surgical intervention, so we can only assume the main cause of this instability and failure. If there was a failure of the polyethylene's locking mechanism, the consequences would appear relatively soon and certainly not 17 years after surgery as in our case [4]. So, we strongly believe that the cause of the failure in our case was the excessive wear of the ultra-high molecular weight polyethylene (UHMWPE). Physicians and especially orthopedic surgeons should be aware that the catastrophic wear of a total hip arthroplasty often imitates a late onset instability. Radiograms in such cases require increased suspicion to realize the cause of the dislocation. The bubble sign [9] is the enlargement of the joint cavity due to the hypersensitivity reaction to the metal debris and polyethylene particles. This radiographic sign should alert orthopedic surgeons. Metal debris shown on the radiograms and excessive osteolysis of the hip joint predispose to catastrophic failure of the arthroplasty and almost exclude a simple arthroplasty dislocation. Catastrophic failure of total hip arthroplasty is multifactorial with the contribution of the patient, surgical and implant-related factors. These factors are the following: age less than 50 years-old, high level of activity, acetabular inclination more than 45 degrees, the difference of more than 18.30 degrees of acetabular inclination between contralateral sides, use of larger femoral heads (32 mm), oxidation of polyethylene due to sterilization, use of non-crosslinked polyethylene and third body wear, especially in ceramic bearings [10, 11]. Follow up is an important independent factor, which prevents excessive catastrophic wear. Until now, there is no consensus concerning the appropriate follow-up of patients who undergo total hip arthroplasty. The current recommendations suggest annual or biennial orthopedic clinical and radiographic examinations [12]. More frequent follow-up is needed in cases of previous revision arthroplasty, previous joint sepsis, osteoporotic bone, very active patients, and surely in symptomatic arthroplasties. In cases with late onset dislocation of total hip arthroplasty, we strongly recommend computed tomography of the joint before reduction attempts, based on the fact that these cases are more complicated than the simple early onset dislocation.

## Conclusions

In the previous decade, UHMWPE usage was the only choice of soft on hard (polyethylene on metal or on the ceramic) bearing surfaces. There is evidence that this type of polyethylene is vulnerable to excessive wear. Orthopedic surgeons should be aware that catastrophic wear of the polyethylene and subsequently the acetabular shell can be presented as a late onset dislocation or protrusion. A high index of suspicion is needed to diagnose this entity early and counsel the patient properly, avoiding any useless reduction attempts. Arthroplasty surgeons should adequately monitor patients who underwent total hip arthroplasty a long time ago, especially with the above-mentioned bearing surfaces. 

## References

[1] Learmonth ID, Young C, Rorabeck C (2007). The operation of the century: total hip replacement. Lancet.

[2] Kurtz S, Ong K, Lau E, Mowat F, Halpern M (2007). Projections of primary and revision hip and knee arthroplasty in the United States from 2005 to 2030. J Bone Joint Surg Am.

[3] Healy WL, Iorio R, Clair AJ, Pellegrini VD, Della Valle CJ, Berend KR (2016). Complications of total hip arthroplasty: standardized list, definitions, and stratification developed by the hip society. Clin Orthop Relat Res.

[4] Joyce C, Harwood JL, Glassman AH (2017). Catastrophic failure of an acetabular total hip arthroplasty component mimicking a posterior dislocation. Arthroplast Today.

[5] Malkani AL, Ong KL, Lau E, Kurtz SM, Justice BJ, Manley MT (2010). Early- and late-term dislocation risk after primary hip arthroplasty in the Medicare population. J Arthroplasty.

[6] Kunutsor SK, Barrett MC, Beswick AD (2019). Risk factors for dislocation after primary total hip replacement: a systematic review and meta-analysis of 125 studies involving approximately five million hip replacements. Lancet Rheumatol.

[7] Pulido L, Restrepo C, Parvizi J (2007). Late instability following total hip arthroplasty. Clin Med Res.

[8] Glyn-Jones S, Isaac S, Hauptfleisch J, McLardy-Smith P, Murray DW, Gill HS (2008). Does highly cross-linked polyethylene wear less than conventional polyethylene in total hip arthroplasty?. J Arthroplasty.

[9] Su EP, Callander PW, Salvati EA (2003). The bubble sign: a new radiographic sign in total hip arthroplasty. J Arthroplasty.

[10] Eggli S, Z’Brun S, Gerber C, Ganz R (2002). Comparison of polyethylene wear with femoral heads of 22 mm and 32 mm: A prospective, randomised study. J Bone Jt Surg.

[11] Manzano G, Levin RAC, Mayor MB, Schwarzkopf R (2014). Catastrophic failure of the acetabular polyethylene liner in ceramic-on-polyethylene total hip arthroplasty. J Orthop case reports.

[12] Teeny SM, York SC, Mesko JW, Rea RE (2003). Long-term follow-up care recommendations after total hip and knee arthroplasty. J Arthroplasty.

